# Intestinal *Ralstonia pickettii* augments glucose intolerance in obesity

**DOI:** 10.1371/journal.pone.0181693

**Published:** 2017-11-22

**Authors:** Shanthadevi D. Udayappan, Petia Kovatcheva-Datchary, Guido J. Bakker, Stefan R. Havik, Hilde Herrema, Patrice D. Cani, Kristien E. Bouter, Clara Belzer, Julia J. Witjes, Anne Vrieze, Noor de Sonnaville, Alice Chaplin, Daniel H. van Raalte, Steven Aalvink, Geesje M. Dallinga-Thie, Hans G. H. J. Heilig, Göran Bergström, Suzan van der Meij, Bart A. van Wagensveld, Joost B. L. Hoekstra, Frits Holleman, Erik S. G. Stroes, Albert K. Groen, Fredrik Bäckhed, Willem M. de Vos, Max Nieuwdorp

**Affiliations:** 1 Department of Vascular Medicine, Academic Medical Center, Amsterdam, The Netherlands; 2 Wallenberg Laboratory, University of Gothenburg, Sahlgrenska University Hospital, Gothenburg, Sweden; 3 Université catholique de Louvain, WELBIO (Walloon Excellence in Life sciences and BIOtechnology), Louvain Drug Research Institute, Brussels, Belgium; 4 Laboratory of Microbiology, Wageningen University, Wageningen, The Netherlands; 5 Department of Internal Medicine, Academic Medical Center, Amsterdam, The Netherlands; 6 Diabetes Center, Department of Internal medicine, VU University Medical Center, Amsterdam, The Netherlands; 7 ICAR, VU University Medical Center, Amsterdam, The Netherlands; 8 Department of Surgery, Flevo Hospital, Almere, The Netherlands; 9 Department of Surgery, Sint Lucas Andreas Hospital, Amsterdam, The Netherlands; 10 Department of Pediatrics, University Medical Center Groningen, University of Groningen, Groningen, The Netherlands; 11 Novo Nordisk Foundation Center for Basic Metabolic Research, Section for Metabolic Receptology and Enteroendocrinology, Faculty of Health Sciences, University of Copenhagen, Copenhagen, Denmark; 12 RPU Immunobiology, University of Helsinki, Helsinki, Finland; Technische Universitat Dresden, GERMANY

## Abstract

An altered intestinal microbiota composition has been implicated in the pathogenesis of metabolic disease including obesity and type 2 diabetes mellitus (T2DM). Low grade inflammation, potentially initiated by the intestinal microbiota, has been suggested to be a driving force in the development of insulin resistance in obesity. Here, we report that bacterial DNA is present in mesenteric adipose tissue of obese but otherwise healthy human subjects. Pyrosequencing of bacterial 16S rRNA genes revealed that DNA from the Gram-negative species *Ralstonia* was most prevalent. Interestingly, fecal abundance of *Ralstonia pickettii* was increased in obese subjects with pre-diabetes and T2DM. To assess if *R*. *pickettii* was causally involved in development of obesity and T2DM, we performed a proof-of-concept study in diet-induced obese (DIO) mice. Compared to vehicle-treated control mice, *R*. *pickettii*-treated DIO mice had reduced glucose tolerance. In addition, circulating levels of endotoxin were increased in *R*. *pickettii*-treated mice. In conclusion, this study suggests that intestinal *Ralstonia* is increased in obese human subjects with T2DM and reciprocally worsens glucose tolerance in DIO mice.

## Introduction

The worldwide epidemic of obesity, which is a major risk factor for insulin resistance, drives the development of common medical conditions such as type 2 diabetes mellitus (T2DM), dyslipidaemia and cardiovascular disease [1]. The development of obesity and T2DM is complex and is driven by both environmental and genetic factors [2]. Obesity-induced inflammatory changes in white adipose tissue have been postulated to play a crucial part in the pathophysiology of obesity and T2DM. Although the majority of our fat depot is located in subcutaneous adipose tissue, approximately 10–20% of the total adipose tissue mass is located intra-abdominally [3]. Especially mesenteric visceral adipose tissue inflammation is linked to insulin resistance reflected in reduced plasma adiponectin levels, which are associated with development of insulin resistance [4] and macrophage influx [5]. In turn, insulin resistance correlates with upregulation of visceral adipose genes involved in innate immunity and inflammation [5].

An increasing body of evidence suggests that the composition of the intestinal microbiota is related to energy intake and obesity [6] and to the development of chronic low-grade inflammation and insulin resistance [7, 8]. This is further supported by data suggesting that (postprandial) endotoxins derived from Gram-negative intestinal bacteria are involved in chronic low-grade inflammation and insulin resistance [9–11]. Indeed, the degree of endotoxemia was found to predict insulin resistance and development of T2DM in otherwise healthy obese subjects [12] through a process that is thought to stem from impaired gut barrier function [13]. Murine studies showed that macrophages in mesenteric adipose tissue indeed contain bacterial DNA that originates from the intestine [14]. However, it remains to be proven that specific intestinal bacteria are indeed causative in the pathogenesis of insulin resistance [15].

Here, we report that bacterial 16S rDNA, including that of the Gram-negative species *Ralstonia*, can be identified in mesenteric visceral adipose tissue of human obese subjects that were otherwise healthy. Interestingly, in a separate cohort of obese subjects with T2DM, fecal abundance of *Ralstonia pickettii* was increased compared to non-diabetic obese controls. To assess a potential causal role of *R*. *pickettii* in development of a diabetes-like phenotype in an obese model system, we treated diet-induced obese (DIO) mice with *R*. *pickettii* for four weeks. Interestingly, *R*. *pickettii*-treated DIO mice had reduced glucose tolerance compared to glycerol treated controls.

## Results

### Identification of *Ralstonia* bacterial DNA in mesenteric visceral adipose tissue from obese individuals

**Fig 1:**
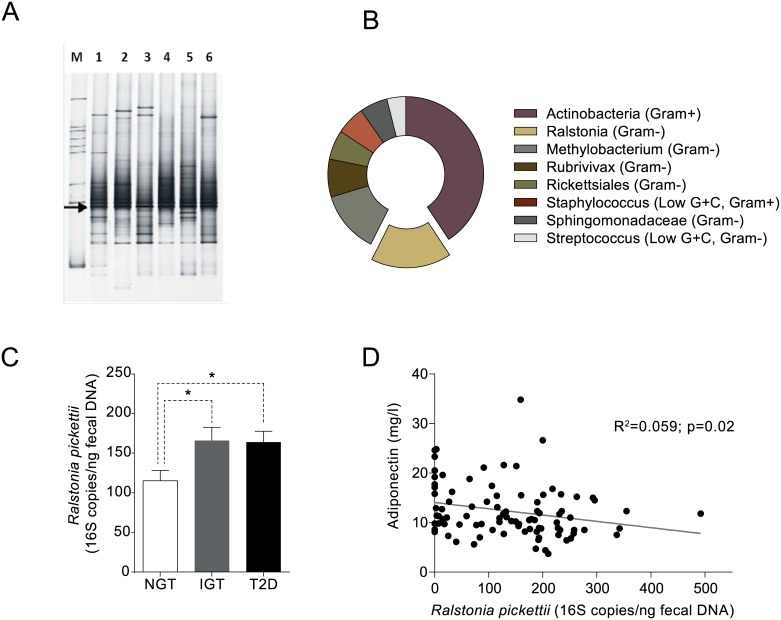
*Ralstonia pickettii* levels correlate with insulin resistance and T2DM in obese subjects. (A) Bacterial DNA is present in mesenteric-visceral adipose tissue from otherwise healthy obese subjects that underwent laparoscopic surgery. Each lane depicts bacterial amplicons in a single mesenteric adipose tissue specimen of a subset of 6 patients. Arrow depicts the dominant amplicon of *Ralstonia* spp. as identified by Sanger sequencing of isolated bands. M = standard. (B) Pyrosequencing revealed presence of different species (percentage of total bacterial DNA) in human mesenteric visceral adipose tissue specimen (n = 12 subjects) with *Ralstonia* spp. being the most abundant Gram-negative bacteria. (C) Fecal 16S rRNA *R*. *pickettii* levels in obese postmenopausal women with normal glucose tolerance (NGT) (n = 42), impaired glucose tolerance (IGT) (n = 45) and type 2 diabetes mellitus (T2DM) (n = 47). (D) Correlation between fecal *R*. *pickettii* and plasma adiponectin in obese postmenopausal women with NGT, IGT and T2DM (population mixed in this figure). Error bars are represented as mean ± SEM. Mann-Whitney U testing (two sided) was performed to analyze the difference between clinical groups (C) and Spearman rank test (two sided) was used to calculate correlation coefficients (D). P-values < 0.05 (indicated by *) were considered statistically significant (using GraphPad Prism 5.1 and SPSS).

Bacterial 16S rDNA was PCR amplified from DNA isolated from human mesenteric visceral adipose tissue biopsies, whereas PCR amplification from omental or subcutaneous adipose tissue biopsies barely yielded any 16S rDNA amplicons. Amplicons from DNA isolated from mesenteric adipose tissue were subjected to denaturing gradient gel electrophoresis (DGGE) profiling (Fig 1A). Subsequent Sanger sequencing identified that the dominant band showed highest similarity to Ralstonia spp. Pyrosequencing analysis of bar-coded 16S rDNA amplicons obtained from the same DNA, identified seven bacterial genera in the mesenteric visceral adipose tissue from obese humans. *Actinobacteria* was the most prevalent Gram-positive and *Ralstonia* the most prevalent Gram-negative bacteria (Fig 1B). Considering emerging data in the field suggesting that endotoxin derived from Gram-negative bacteria is involved in metabolic endotoxemia and reduced glucose tolerance [9, 14, 16], for this project we focused on the role of *Ralstonia* in glucose homeostasis.

The *Ralstonia* genus belongs to the Proteobacteria phylum and *Burkholderiales* order, and comprises flagellated facultative anaerobic Gram-negative rod-shaped bacteria that are predominantly found in soil and water. Four species of *Ralstonia* (*R*. *insidiosa*, *R*. *eutropha*, *R*. *mannitolilytica and R*. *pickettii*) are known to reside in the human intestinal tract, with *R*. *pickettii* being most frequently associated with human infections [15].

### Increased levels of fecal *R*. *pickettii* in patients with IGT or T2DM

Based on previous data regarding the association between altered intestinal microbiota and insulin resistance, we thus tested the hypothesis whether fecal *R*. *pickettii* levels could classify subjects with normal glucose tolerance (NGT), impaired glucose tolerance (IGT) and T2DM in a cohort of otherwise healthy subjects [7, 17]. Interestingly, fecal *R*. *pickettii* levels were significantly increased in IGT and T2DM subjects compared to NGT controls (Fig 1C). Furthermore, fecal *R*. *pickettii* levels correlated significantly (r = 0.059, p = 0.02) with plasma adiponectin in IGT and T2DM subjects (Fig 1D). Based on these associative data in obese T2DM human subjects, we questioned if *R*. *pickettii* could place a causal driving role in development of insulin resistance in a rodent model for obesity and insulin resistance.

### Metabolic effects of *R*. *pickettii* gavage in diet induced obesity (DIO) mice

To examine potential causality of *R*. *pickettii* in development of obesity and insulin resistance, mice were fed a high-fat diet (HFD) (60% Kcal) for eight weeks. DIO-mice were then gavaged daily with heat-inactivated (HI) or living *R*. *pickettii* (10^6^ CFU in 10% glycerol in PBS, final volume 100ul) for four weeks. 10% glycerol in PBS (glycerol) was used as control treatment.

**Fig 2:**
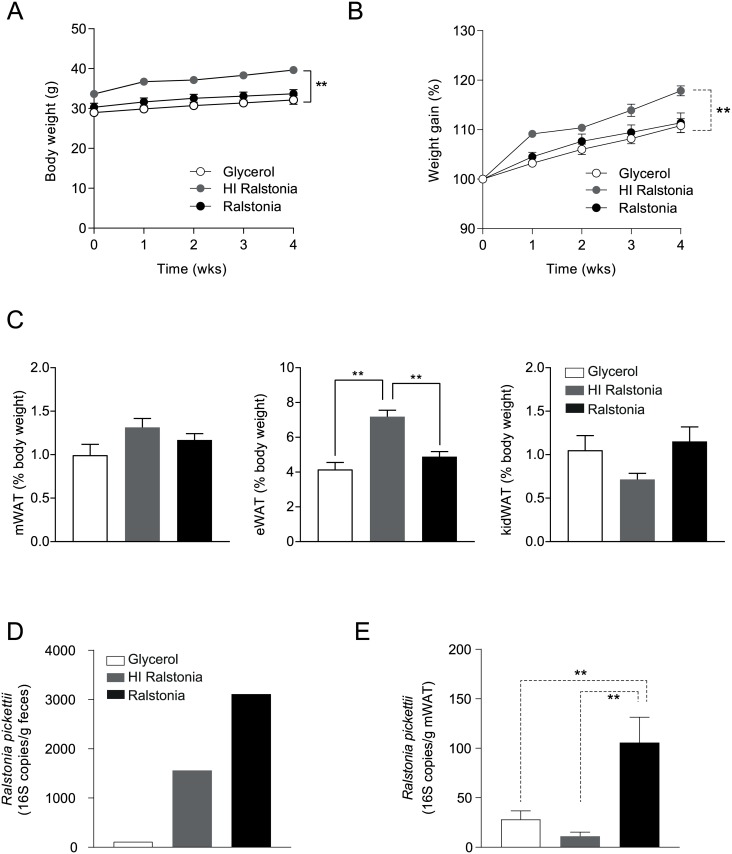
*Ralstonia pickettii* augments weight gain in DIO mice. Diet-induced obese (DIO) C57Bl6 mice received 10E6 CFU heat-inactivated (HI)- or *R*. *pickettii* daily by means of oral gavage for four weeks. Glycerol was used as control. (A) Absolute body weight (g) during intervention time. HI *R*. *pickettii* group had higher starting body weight compared to glycerol and *R*. *pickettii* treated mice and gained more weight throughout the gavage experiment. (B) Relative (%) weight gain of glycerol, heat-inactivated (HI)- and *R*. *pickettii*-treated mice. HI-*R*. *pickettii*-treated mice gained more weight compared to glycerol- and *R*. *pickettii*-treated counterparts. (C) Relative weight (as % of body weight at time of termination) of mesenteric white adipose tissue (mWAT); epididymal white adipose tissue (eWAT) and kidney white adipose tissue (kWAT). eWAT weight was higher in HI *R*. *pickettii*-treated mice compared to glycerol and *R*.*pickettii*-treated mice. (D) qPCR analysis of *R*. *pickettii* DNA abundance per gram feces (per cage of mice) treated with glycerol, HI *R*. *pickettii* and *R*. *pickettii*. (E) qPCR analysis of *R*. *pickettii* DNA abundance per gram mesenteric white adipose tissue (mWAT) of mice treated with glycerol, HI *R*. *pickettii* and *R*. *pickettii*. N = 10 mice per group. Error bars are represented as mean ± SEM; p values were determined by Mann-Whitney U test or two-way ANOVA testing with Bonferroni post-test for multiple-comparison analysis (for weight gain). P-values < 0.05 (indicated by *) or < 0.01 (indicated by **) were considered statistically significant (using GraphPad Prism 5.1 and SPSS).

During the four-week treatment period, weight gain in HI-*R*. *pickettii*-treated DIO mice was increased compared to glycerol- and *R*. *pickettii* treated controls (Fig 2A). It is important to point out though, that HI-*R*. *pickettii*-treated DIO mice had slightly higher body weight at the start of the treatment period. Although we cannot fully explain this discrepancy, it might in part contribute to the increased relative weight gain (Fig 2B) upon HI *R*. *pickettii* treatment. The epididymal white adipose tissue (eWAT) compartment was significantly increased in HI-*R*. *pickettii*-treated mice compared to glycerol and *R*. *pickettii*-treated mice whereas mesenteric and kidney WAT compartments did not differ between groups (Fig 2C). *R*. *pickettii* content was increased in feces of HI-*R*. *pickettii* and *R*. *pickettii*-treated mice compared to glycerol-treated controls (Fig 2D). In contrast, *R*. *pickettii* DNA was not increased in mesenteric white adipose tissue (mWAT) of HI-*R*. *pickettii* treated mice compared to glycerol controls, whereas *R*. *pickettii* treated mice had increased levels of *R*. *pickettii* DNA in this adipose tissue compartment (Fig 2E). This suggests that live bacteria may be required for translocation from the gut consistent with a previous finding (14).

**Fig 3:**
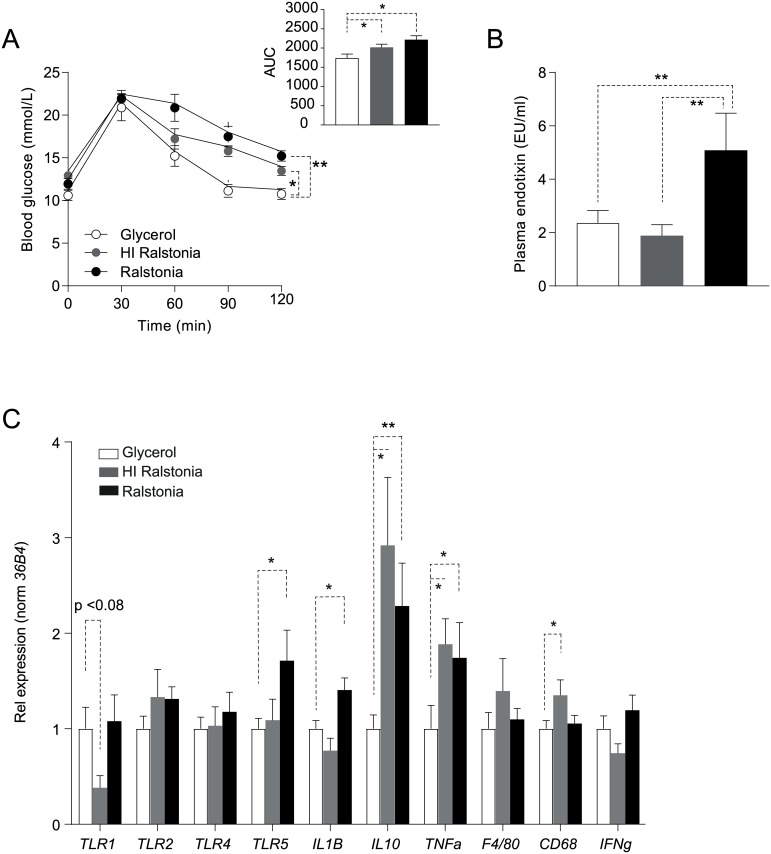
*Ralstonia pickettii* supplementation reduces glucose tolerance and augments inflammatory tone in DIO mice. (A) Oral glucose tolerance testing (OGTT) revealed that (HI)-*R*. *pickettii* treatment decreased glucose tolerance compared to glycerol treatment in DIO-mice. Area under the curve (AUC) is represented in the insert. (B) Plasma endotoxin levels (EU/ml) were increased in *R*. *pickettii*-treated mice compared with glycerol and HI *R*. *pickettii*-treated counterparts. (C) Relative mRNA expression of *Tlr1*, *Tlr2*, *Tlr4*, *Tlr5*, *IL1B*, *IL10*, *TNFα*, *F4/80*, *CD68* and *IFNγ* in mesenteric white adipose tissue (mWAT) of mice treated with glycerol, HI *R*. *pickettii* and *R*. *pickettii*. Gene expression was normalized using *36B4* as a housekeeping gene. N = 10 mice per group. Error bars are represented as mean ± SEM; p values were determined by Mann-Whitney U test or two-way ANOVA testing with Bonferroni post-test for multiple-comparison analysis (for OGTT). P-values < 0.05 (indicated by *) or < 0.01 (indicated by **) were considered statistically significant (using GraphPad Prism 5.1 and SPSS).

Oral glucose tolerance testing (OGTT) in week three of the treatment period revealed that clearance of glucose from the circulation was reduced in HI- and *R*. *pickettii*-treated DIO mice (Fig 3A). To assess if HFD feeding was a prerequisite to reduce glucose tolerance following four weeks of *R*. *pickettii* treatment, lean, chow-fed mice (age-matched with DIO mice) were gavaged daily with *R*. *pickettii* (10^6^ CFU in 10% glycerol in PBS, final volume 100ul) for four weeks. Importantly, lean mice were not susceptible to weight gain and reduced glucose tolerance during the four week treatment period (S1A and S1B Fig).

Metabolic endotoxemia, a process resulting from translocation of endotoxic compounds (*e*.*g*., LPS) of Gram-negative intestinal bacteria, was first described based on the association between alterations in intestinal microbiota composition, circulating levels of the bacterial cell membrane component LPS and onset of T2DM (9). To assess if *R*. *pickettii* administration affected circulating endotoxin levels, we analyzed endotoxin levels in (HI) *R*. *pickettii*-treated DIO (Fig 3B) and lean (S1C Fig) mice. In DIO mice, endotoxin levels were significantly increased in the live *R*. *pickettii*-treated group compared to glycerol treated controls. Interestingly, HI-*R*. *pickettii*-treatment did not increase circulating endotoxin levels: endotoxin levels were comparable to levels in glycerol-treated DIO mice (Fig 3B). Although treatment with live *R*. *pickettii* increased endotoxin levels compared to treatment with HI-*R*. *pickettii* in lean mice, this difference did not reach statistical significance (S1C Fig). In line, fecal *R*. *pickettii* DNA content (S1D Fig) was higher only in live *R*. *pickettii*-treated lean mice compared to the HI *R*. *pickettii* and glycerol treated controls. Despite similar daily CFU doses, HI-*R*. *Pickettii*-treated DIO mice had reduced levels of circulating endotoxin compared to live *R*. *pickettii*-treated mice and this group gained more weight and had reduced glucose tolerance compared to glycerol-treated mice. This indicates that potential translocation and endotoxemia are not a prerequisite to develop reduced glucose tolerance upon Ralstonia administration.

Toll-like receptors (TLR) are pattern recognition receptors (PPRs) that interact with bacterial cell wall components and can subsequently induce an inflammatory response. To investigate the effect of *R*. *pickettii* administration on mWAT inflammation, gene expression levels of *Tlr1*, *Tlr*2, *Tlr4* and *Tlr5* were measured using qPCR (Fig 3C). *Tlr5* expression was significantly upregulated in mWAT of DIO-mice treated with *R*. *pickettii* compared to HI- *R*. *pickettii* or glycerol-treated controls. In addition, expression of interleukins (*IL) 1B* and *Il10* were significantly enhanced and indicative of activation of inflammatory pathways in mWAT of *R*. *pickettii*-treated DIO-mice. *TNFa*, *F4/80*, *CD68 and CD14a* were unaffected by (HI) *R*. *pickettii*-treatment. As control, gene expression of *Tlr1*, *Tlr2*, *Tlr4*, *Tlr5*, *IL1B*, *IL10*, *TNFα*, *F4/80*, *CD68* and *IFNγ* was assessed in epididymal white adipose tissue (eWAT, S1E Fig). In contrast to mWAT, expression levels of assessed genes were mainly unaffected in eWAT. TNFα expression, however, like TNFα expression in mWAT, was significantly increased in (HI)-*R*. *pickettii*- treated mice compared to glycerol-treated controls. To assess if circulating TNFα levels were affected, we performed ELISA analysis in plasma. Circulating TNFα levels were below detection limit in all study groups.

Error bars are represented as mean ± SEM; p values were determined by Mann-Whitney U test or two-way ANOVA testing with Bonferroni post-test for multiple-comparison analysis (for weight gain and OGTT). P-values < 0.05 (indicated by *) or < 0.01 (indicated by **) were considered statistically significant (using GraphPad Prism 5.1 and SPSS).

## Discussion

The development of insulin resistance and type 2 diabetes mellitus is associated with low-grade inflammation, or metabolic endotoxemia [10, 12, 16]. Although there is much debate as to whether intestinal microbiota composition is a causal factor or just a bystander in development of T2DM in humans [7, 8, 18, 19], mesenteric visceral adipose tissue inflammation is a well-known pathophysiological driver of insulin resistance [2]. Others have shown that Gram-negative flagellin-bearing pathogens, including *Ralstonia* are present in feces of human T2DM patients [16, 20]. In agreement with these observations we here demonstrate that DNA from Gram-negative *Ralstonia* species resides in human intestine but also in mesenteric visceral adipose tissue. Moreover, *R*. *pickettii* fecal concentrations were associated with impaired glucose tolerance and T2DM in human obese subjects. Furthermore, fecal *R*. *pickettii* levels correlated with plasma adiponectin levels as marker for impaired metabolic control.

Our proof-of-concept studies in mice suggested that intestinal bacterial strains like *R*. *pickettii* may indeed be causally linked to the pathophysiology of insulin resistance in obesity. *R*. *pickettii* inoculation reduced glucose tolerance and increased markers of mesenteric visceral adipose tissue inflammation in DIO mice. Interestingly, heat-inactivated bacteria generated similar effects as viable bacteria. Lean, chow-fed mice did not develop these pathologies when challenged with this bacterium. A HFD diet therefore seems to be a prerequisite for *R*. *pickettii*-mediated augmentation of metabolic derangements.

Our findings align with recent studies using murine conditional knockout models that suggest a distinct role for the intestinal bacterial pathogens and epithelial pattern recognition receptors in the development of insulin resistance [21–25]. The underlying mechanisms, however, remain to be studied but could be mediated by intestinal adaptive immune cells such as Innate Lymphoid Cells (ILC) [26, 27] or via enhanced B cell-mediated IgA antibody production against pathogens such as *Ralstonia* [28]. A potential relation between inflammatory tone, the innate immune system and TLRs has been implicated in the development of murine obesity and T2DM [5, 29, 30]. On the other hand, intestinal pathogens catabolize mucosal carbohydrates during their expansion that could subsequently enhance bacterial translocation [31]. Bacterial translocation has been defined as the passage of viable bacteria from the gastrointestinal tract to otherwise sterile peripheral tissues. This translocation potentially occurs via mesenteric lymph nodes and then to peripheral organs [32]. Recent data obtained from studies in humans have suggested that bacteria might be able to directly translocate from the intestinal wall to mesenteric adipose tissue via the circulation [33].

Our study has certain limitations. First, as *Ralstonia* spp. can be found in different environments including soil and (drinking) water [34], we cannot fully exclude potential contamination [35]. However, as we could not identify bacterial (*Ralstonia*) DNA in human omental and subcutaneous adipose tissue or in our controls, this seems to be less plausible. Moreover, the fact that *R*. *pickettii* inoculation reduced glucose tolerance in mice reduces the likelihood that *R*. *pickettii* effects are merely due to contamination. Second, it is currently unknown whether bacterial DNA detected in mesenteric adipose tissue is derived from alive or dead bacteria and studies using labelled bacteria are needed to study *in vivo* bacterial translocation. In addition, it remains to be determined whether bacterial DNA is equally present in all mesenteric adipose tissue depots along the human gastrointestinal tract and what mechanism underlies potential *Ralstonia* translocation. Our findings that HI-*R*. *pickettii*-treated mice have reduced circulating endotoxin levels and reduced 16S rDNA content in mesenteric adipose tissue suggests that in part alive bacteria are required in order to breach the gut-epithelial lining and reach the extra-intestinal compartment. Inflammatory markers in mesenteric adipose tissue of HI- or live *R*. *pickettii*-groups, however, were increased to similar extend for some (*i*.*e*., IL10, TNFα and CD68) but not all (*i*.*e*., TLR5 and IL1ββ) genes. If and how these differences in inflammatory expression patterns are related to potential bacterial translocation remains to be determined. Nevertheless, both HI-*R*. *pickettii* and active *R*. *pickettii*-treated mice had reduced glucose tolerance compared to glycerol-treated controls. Although we have not addressed this option, we speculate that increased levels of HI- *R*. *pickettii* might be sensed by the local intestinal immune system, thereby affecting inflammatory tone and augmenting glucose intolerance [36].

In conclusion, this proof-of-concept study shows that specific gram negative intestinal bacterial like *R*. *pickettii* are associated with the pathophysiology of insulin resistance in obesity. Our data support the preliminary hypothesis that bacterial translocation of these bacterial strains might be involved in the development of insulin resistance. Disentangling such a specific signature of intestinal microbiota involved in insulin resistance shifts might help to apply approaches aiming to better predict loss of insulin sensitivity and design targeted microbiota-based interventions in obese humans.

## Materials and methods

### Participants

Caucasian subjects (Fig 1A and 1B) (male/postmenopausal females, scheduled for elective laparoscopic cholecystectomy) were screened by the attending surgeon (n = 12 individuals included). Inclusion criteria were: age between 18–75 years and body-mass index (BMI) between 25–40 kg/m^2^. Exclusion criteria were: malignancy, diagnosed T2DM, chronic inflammatory disease and use of probiotics and/or antibiotics in the past three months. Written informed consent was obtained from all subjects. The study was approved by the AMC Ethics committee and conducted at the Flevo hospital (Almere, The Netherlands), Sint Lucas Andreas hospital (Amsterdam, The Netherlands) and Academic Medical Center (Amsterdam, The Netherlands), in accordance with the Declaration of Helsinki. Participants could continue their own diet, but were asked to fill out a week-long online nutritional diary (www.dieetinzicht.nl) to monitor caloric intake. Prior to surgery, anthropometric measurements were taken and a fasted blood sample was taken to determine levels of metabolic parameters in plasma. (see S1 Table).

The DIWA study (Fig 1C and 1D) included overweight women (average age 70 years old, BMI 25.8 to 28 kg/m2) with either normal glucose tolerance (NGT), impaired glucose tolerance (IGT) or type 2 diabetes mellitus (T2DM). Exclusion criteria were chronic inflammatory disease and treatment with antibiotics during the preceding three months. Further details about this cohort have been described elsewhere [7, 17]. All subjects gave informed consent and provided a fresh morning stool sample.

### Animals

Male C56BL6/J mice were obtained from Charles River Laboratories. Mice were randomly allocated in treatment groups. Mice were fed a standard laboratory chow diet (Research Diets Inc., USA) or a high-fat diet (HFD) (60% Kcal fat, D12492, Research Diets Inc., USA) as indicated in the manuscript. Dietary components of the HFD are depicted in Table 1.

**Table 1:** Dietary components of the HFD.

	**gm%**	**kcal%**
Protein	26.2	20
Carbohydrate	26.3	20
Fat	34.9	60
**Total kcal/gm**	**5.24**	
	**gm**	**kcal**
Casein, 30 Mesh	200	800
L-Cysteine	3	12
Corn Starch	0	0
Maltodextrin 10	125	500
Sucrose	68.8	275.2
Cellulose, BW200	50	0
Soybean Oil	25	225
Lard	245	2205
Mineral Mix S10026	10	0
DiCalcium Phosphate	13	0
Calcium Carbonate	5.5	0
Potassium Citrate, 1 H2O	16.5	0
Vitamin Mix V10001	10	40
Choline Bitartrate	2	0
FD&C Blue Dye #1	0.05	0
**Total**	**773.85**	**4057**

Diet-induced obesity (DIO) was realised by feeding mice a HFD for eight weeks starting at four weeks of age. Mice were housed in a constant 12-hour light-dark cycle with controlled temperature and humidity and were given access to food and water *ad libitum*.

Starting at the age of 13 weeks, *Ralstonia pickettii* (DSM 6297, Deutsche Sammlung von Mikroorganismen und Zellkulturen) was administered daily for four weeks by oral gavage (10^6^ CFU in 10% glycerol in PBS, final volume 100ul). Heat-inactivated (10 min at 70°C) *R*. *picketti* (10^6^ CFU in 10% glycerol- PBS, final volume 100ul) and glycerol (10% in PBS, final volume 100ul) were used as controls. Heat-inactivation fully impaired the ability of *R*. *pickettii* to grow (S2 Fig). Viability and purity of all *R*. *pickettii* stored at -80°C was tested up to 12 months by culture and sequencing. Weight gain and food intake were monitored throughout the treatment period. Feces was collected per cage (n = 5 mice per cage) in week three of treatment.

Oral glucose tolerance tests (OGTT) were performed in week three of the treatment period. Mice were fasted for 4 hours and blood glucose levels were measured from the tip of the tail. Mice received an oral bolus of D-glucose (2 g/kg bodyweight in 200ul sterile saline) and blood glucose levels were subsequently measured at t = 30, 60, 90, and 120 minutes.

After four weeks of *R*. *pickettii-* or control-treatment, mice were terminated by cardiac puncture under sodium pentobarbital anesthesia. Blood was collected in EDTA-coated tubes and was kept on ice until centrifugation (8,000xg, 4°C, 20min). Plasma was aliquoted and used for analysis immediately or stored at -80°C. Organs and tissues including mesenteric adipose tissue (aligning the colon transversum) were quickly excised under sterile conditions, snap-frozen in liquid nitrogen and stored at -80°C until further analysis.

All animal experiments were conducted in accordance with the principles of the “Guide to the Care and Use of Experimental Animals” and were prospectively approved by the Institutional Animal Care and Use Committee ("Dierexperimentencommissie (DEC) of the Academic Medical Center (AMC) in Amsterdam).

### Bacterial DNA isolation and sequencing

Genomic DNA of both prokaryotic and eukaryotic origin was isolated from biopsies according to the phenol-choloform method as described by Zoetendal *et al*. [37]. In short, a standardized amount of fat tissue was treated with a mix of SDS and proteinase K at 55°C and homogenized by mechanical disruption using zirconium glass beads (1mm) in the FAST Prep-24 (MP Biomedical) in the presence of phenol. The genomic DNA was extracted using a series of phenol/chloroform extractions and precipitated in the presence of absolute ethanol.

The prokaryotic fraction was studied using a range of 16S rRNA specific primers and assays. Full-length 16S rDNA amplicons were generated using PCR by using primers Bact-27F (5’GTTTGATCCTGGCTCAG-3’) and Prok-1392R (5’GCCCGGGAACGTATTCACCG-3’). The PCR conditions have been described by Rajilic-Stojanovic *et al*. [38]. The resulting amplicons were purified and used as input for a nested PCR using primers 968-GC-F and 1392, generating fragments fit for a diversity analysis by denaturing gradient gel electrophoresis (DGGE) profiling using conditions described by Heilig *et al*. [39]. Importantly, no amplicons were obtained from control PCRs with water and buffers. The dominant band appearing in the DGGE analyses was subcloned from the DGGE amplicon in a pGEM-T easy vector (Promega, Leiden, The Netherlands) and transformed into Stratagene *E*. *coli* XL-1 Blue competent cells (Agilent Technologies, Amstelveen, The Netherlands) according to the manufacturers’ specifications. Clones containing the correct insert that migrated to the same position as the dominant band in the DGGE gel were subjected to Sanger Next Generation Sequence analysis (GATC Biotech, Konstanz, Germany). Sequences were identified by performing a BLAST search (http://blast.ncbi.nlm.nih.gov/Blast.cgi). Sequence analysis of the dominant band appearing in DGGE showed highest similarity to sequences of *Ralstonia* species.

Moreover, the genomic DNA was subjected to 454-pyrosequencing of the V4-V6 region of the 16S rRNA (using primers 520F 5'- AYT GGG YDT AAA GNG -3' and 1100R 5'- GGG TTN CGN TCG TTG -3'). The quality-controlled reads (normalized to at least 1000 reads per sample) were processed through the QIIME pipeline [40]. To quantify the *Ralstonia*-spp. bacteria, we performed a qPCR in the fecal DNA and mWAT DNA samples [41]. Samples were analyzed in a 25-μl reaction mix consisting of 12.5 μl 1xSYBR Green Master Mix buffer (Thermo Scientific, Waltham, Massachusetts, USA), water, 0.2 μM of each primer and 5 μl of template of genomic DNA extracted from feces or mWAT. Standard curve of 16S rRNA PCR product of *Ralstonia pickettii* was created using serial 10-fold dilution of purified full length 16S rDNA PCR product. The qPCR primers were based on *R*. *pickettii* (F’: ATGATCTAGC-TTGCTAGATTGAT; R’: ACTGATCGTCGCCTTGGTG). Data are expressed as copies of 16S rDNA *Ralstonia* compared to total bacterial DNA [42].

### Plasma endotoxin measurement

Blood LPS endotoxin activity was measured using Endosafe-MCS (Charles River Laboratories, Lyon, France) based on the Limulus amaebocyte Lysate (LAL) kinetic chromogenic methodology that measures color intensity directly related to the endotoxin concentration in a sample. Plasma was diluted 1/10 with endotoxin free buffer (Charles River Laboratories) to minimize interferences in the reaction and heated for 15 min at 70°C. Each sample was diluted with endotoxin-free LAL reagent water (Charles River Laboratories) and treated in duplicate. Two spikes for each sample were included in the determination. All samples have been validated for the recovery and the coefficient variation. The lower limit of detection was 0.005 EU/ml [43].

#### Quantitative Real time PCR

Mouse (mesenteric and epidydimal) adipose tissue sections were homogenized using tissue-magnaLyzer (Roche, Switzerland). Total RNA was extracted using Tri-pure reagent (Roche). cDNA was prepared by reverse transcription of 1μg total RNA using a reverse transcription kit (BioRad, USA). Real-time qPCR was performed using Sensifast SYBR master mix (GC biotech). Gene-specific intron-exon boundary spanning primers were used and all the results were normalized to the house keeping gene *36B4*. All samples were analyzed in duplicate and data were analyzed according to the 2^ΔΔCT^ method.

### Statistical analysis

Mann Whitney tests (two sided) were used to analyze the difference between (clinical) groups. Spearman rank test (two sided) was used to calculate correlation coefficients. P-values < 0.05 (indicated by *) or < 0.01 (indicated by **) were considered statistically significant (using GraphPad Prism 5.1 and SPSS).

## Supporting information

S1 Fig Lean mice are protected from *Ralstonia pickettii*-mediated glucose intolerance. Lean, chow-fed C57Bl6 mice received 10E6 CFU (HI) *R*. *pickettii* daily by means of oral gavage for four weeks. Glycerol was used as control. (A) Relative (%) weight gain during four weeks of glycerol, HI *R*. *pickettii* or *R*. *pickettii* administration. (B) Oral glucose tolerance tests (OGTT) in glycerol, HI *R*. *pickettii* or *R*. *pickettii*-treated mice. (C) Plasma endotoxin levels (EU/ml) in 4-hr fasted mice treated with glycerol, HI *R*. *pickettii* and *R*. *pickettii*. (D) qPCR analysis of *R*. *pickettii* DNA abundance per gram feces (per cage of mice) treated with glycerol, HI *R*. *pickettii* and *R*. *pickettii*. (E) Diet-induced obese (DIO) C57Bl6 mice received 10E6 CFU heat-inactivated (HI)- or *R*. *pickettii* daily by means of oral gavage for four weeks. Glycerol was used as control. Relative mRNA expression of *Tlr1*, *Tlr2*, *Tlr4*, *Tlr5*, *IL1B*, *IL10*, *TNFα*, *F4/80*, *CD68* and *IFNγ* in epididymal white adipose tissue (eWAT) of DIO mice treated with glycerol, HI *R*. *pickettii* and *R*. *pickettii*. Gene expression was normalized using *36B4* as a housekeeping gene. N = 10 mice per group. Error bars are represented as mean ± SEM; p values were determined by Mann-Whitney U test or two-way ANOVA testing with Bonferroni post-test for multiple-comparison analysis (for weight gain). P-values < 0.05 (indicated by *) or < 0.01 (indicated by **) were considered statistically significant (using GraphPad Prism 5.1 and SPSS).
(EPS)

S2 Fig Heat-inactivation fully impairs the ability of *R*. *pickettii* to grow. *R*. *pickettii* was heat-inactivated at 70°C for 10min. HI- or live *R*. *pickettii* were streaked onto blood agar plates and grown aerobically at 37°C overnight. Arrows indicate example colonies or *R*. *pickettii*.
(EPS)

S1 Table Anthropometric data of human subjects scheduled for surgery. Data are presented as mean±standard deviation.
(EPS)
