# Correction: Intestinal *Ralstonia pickettii* augments glucose intolerance in obesity

**DOI:** 10.1371/journal.pone.0192339

**Published:** 2018-01-30

**Authors:** The PLOS ONE staff

The eleventh author's name appears incorrectly. The correct name is: Eleonore Susanne Victoria de Sonnaville. The publisher apologizes for the error.

## References

[1] Udayappan SD, Kovatcheva-Datchary P, Bakker GJ, Havik SR, Herrema H, Cani PD, et al (2017) Intestinal *Ralstonia pickettii* augments glucose intolerance in obesity. PLoS ONE 12(11): e0181693 https://doi.org/10.1371/journal.pone.0181693 29166392 10.1371/journal.pone.0181693 PMC5699813

